# Expression of miR-664-3p in Osteosarcoma and Its Effects on the Proliferation and Apoptosis of Osteosarcoma Cells

**Published:** 2019-10

**Authors:** Ye LI, Jie TANG, Yong HU, Yonghai PENG, Junwen WANG

**Affiliations:** 1. Department 3 of Orthopedics, Wuhan Fourth Hospital, Puai Hospital, Tongji Medical College, Huazhong University of Science, Wuhan 430034, P.R. China; 2. Department 2 of Orthopedics, Wuhan Fourth Hospital, Puai Hospital, Tongji Medical College, Huazhong University of Science, Wuhan 430034, P.R. China

**Keywords:** Apoptosis, miR-664-3p, Osteosarcoma, Proliferation, U2-OS

## Abstract

**Background:**

To explore the expression level of miR-664-3p in osteosarcoma and its effects on the proliferation and apoptosis of osteosarcoma cells.

**Methods:**

Specimens of osteosarcoma tissues were collected from 41 cases undergoing surgical treatment in the Orthopedics Department of Wuhan Puai Hospital, Wuhan, China from January 2015 to February 2018. Another 40 cases of normal bone tissue were collected. The expression of miR-664-3p were detected using quantificational real-time polymerase chain reaction. miR-664-3p mimics, miR-664-3p inhibitor and miR-664-3p negative control (NC) were used to transfect U2-OS, which were named as mimics group, inhibitor group and NC group, respectively. MTT assay was adopted to detect the effects of microRNA-664-3p on the proliferation of U2-OS after 24, 48, 72, 96 and 120 hours of transfection. Flow cytometry was applied to measure the apoptosis rate of U2-OS after miR-664-3p transfection. Finally, Western Blot was employed to detect the expression of proteolipid protein 2 (PLP2).

**Results:**

The total apoptosis rate of cells in the inhibitor group was obviously higher than those in the mimics group and the NC group (*P*<0.001). The relative expression level of PLP2 in the inhibitor group was significantly lower than those in the mimics group and the NC group (*P*<0.001).

**Conclusion:**

MiR-664-3p may be involved in the occurrence and development of osteosarcoma, and can regulate the proliferation and apoptosis of U2-OS cells, and the expression of PLP2. Besides, miR-664-3p may become a novel molecular biological indicator for the diagnosis, targeted treatment and prognosis assessment of osteosarcoma.

## Introduction

Osteosarcoma, a malignant bone tumor originated from the skeletal system in clinical practice, often occurs in adolescents. The peak age of onset is 16–25 years old. Its incidence rate is approximately 3/1 million, taking up about 56% of all bone tumors (1). Distant metastasis occurs in the early stage of osteosarcoma, and the disease is characterized by high deterioration degree and high metastasis rate. Patients with osteosarcoma often have poor prognosis and a high recurrence rate. Once osteosarcoma recurs, the prognosis of patients will become worse (2–4).

As the medical technology and chemotherapy continuously advance, adjuvant chemotherapy has been widely applied in the treatment of osteosarcoma. The survival period of patients with osteosarcoma can be greatly prolonged by the comprehensive treatment mainly based on limb salvage surgery, and the 5-year survival rate can reach 65%–70% (5). Nevertheless, the survival rate of osteosarcoma patients with distant metastasis and high degree of malignancy is still not optimistic, with the median survival time of only about 20 months. The underlying reason is that osteosarcoma cells have no sensitivity to chemotherapy drugs, which leads to multidrug resistance, thus resulting in insignificant curative effects of chemotherapy (6). Therefore, it is of great importance to carry out in-depth research on osteosarcoma.

miRNAs participate in the occurrence and development of a variety of diseases, including cardiovascular diseases, autoimmune diseases and tumor diseases (7–9). Multiple miRNA molecules show abnormal expression in osteosarcoma tissues and cells, and have close correlations with the invasion, proliferation, apoptosis and drug resistance of osteosarcoma cells (10). As a new-found miRNA in recent years, miR-664-3p is found to be highly expressed in the longevous mice (11).

In this study, the roles of miR-664-3p in the occurrence and development of osteosarcoma and its influence on the proliferation and apoptosis of osteosarcoma cells were explored by measuring the expression of miR-664-3p in osteosarcoma tissues and cells, thus providing reference for the early diagnosis and targeted treatment of osteosarcoma.

## Methods

### General data

Specimens of osteosarcoma tissues were collected from 41 cases undergoing surgical treatment in the Orthopedics Department of Wuhan Puai Hospital, Wuhan, China from January 2015 to February 2018. Normal bone tissues (>5 cm away from the tumor lesion) were collected from another 40 cases. Forty one patients with osteosarcoma consisted of 22 males and 19 females aged 12–46 years old, with an average age of (20.63±4.63) years old.

Inclusion criteria: patients who received no treatment before surgery, who underwent surgical treatment, whose osteosarcoma tissue and normal bone tissue specimens were definitely diagnosed by pathology (12), and whose postoperative tissue specimens were immediately placed in liquid nitrogen for 5 h, followed by cryopreservation at −80 °C. Exclusion criteria: patients with previous mental illness or a family history of mental illness, or those complicated with severe heart, lung, liver or kidney dysfunction.

This study was approved by the Ethics Committee of Wuhan Puai Hospital; both the study subjects and their family members were informed of this study. We provided written informed consent to the patients or their guardians, and the patients signed the informed consent.

### Prediction of miR-664-3p related target genes

**Fig. 1:**
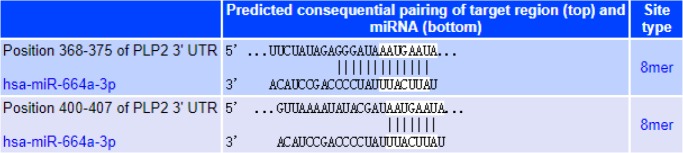
Prediction of miR-664-3p related target genes

TargetScan Release 7.2 online software (http://www.targetscan.org) was applied to perform target gene predictive analysis on miR-664-3p, and the results showed that proteolipid protein 2 (PLP2) may be a potential target gene of miR-664-3p (Fig. 1).

### Main instruments, reagents and materials

7500 fluorescence quantitative polymerase chain reaction (qPCR) instrument (ABI, USA), FACSCalibur flow cytometer (BD, USA), miR-664-3p fluorescence qPCR kit (Shenzhen Huaan Pingkang Biotechnology Co., Ltd.), SYBR Green qPCR Master Mix kit [Thermo Fisher Scientific (China) Co., Ltd.], RNA extraction kit (TRIzol assay) and Moloney Murine Leukemia Virus (MMLV) reverse transcription (RT) kit (Invitrogen, USA), normal human osteoblast line hFOB1.19 as well as osteosarcoma cell lines SOSP-9607 and U2-OS (Medical Laboratory Center of Nanchang University), miR-664-3p mimics, miR-664-3p inhibitor and miR-664-3p negative control (NC) [Tiangen Biotech (Beijing) Co., Ltd.], proteolipid protein 2 (PLP2) (Shanghai Hengfei Biotechnology Co., Ltd., China, bs-12733R-1), HRP-labeled goat anti-mouse second antibody (Beyotime Biotechnology, Shanghai, China, YY-70127), and detection kits for cell proliferation by MTT and for cell apoptosis by Annexin V-FITC (Shanghai Beyotime Biotechnology Institute). Real-time fluorescence qPCR miR-664-3p, and U6 internal reference sequences were designed and synthesized by Annoron (Beijing) Biotechnology Co., Ltd. (Table 1).

**Table 1:** Primer sequences

***Gene***	***Forward primer sequence***	***Reverse primer sequence***
MiR-664-3p	5′-ACACTCCAGCTGGG-TATTCATTTATCCCCAGCC-3′	5′-GCGAGCACA GAATTAATACGAC-3′
U6	5′- CTCGCTTCGGCAGCACA -3′	5′-AACGCT TCACGAATTTGCGT-3′

### Cell culture

hFOB1.19, SOSP-9607 and U2-OS were inoculated into culture dishes, added with Roswell Park Memorial Institute (RPMI) 1640 medium containing 15% fetal bovine serum and 1% penicillin/streptomycin, and incubated in an incubator with 5% CO_2_ at 37°C under constant temperature and humidity. The fluid was exchanged in due time. The cells were subjected to routine digestion and passage with trypsin, and those in the logarithmic growth phase were used for follow-up experiments.

### Cell transfection

Cells in the logarithmic growth phase were inoculated into a 6-well plate at a concentration of 1×10^5^ cells/well, followed by transfection after 60–70% cells were fused. The cells were divided into 3 groups, namely, the mimics group (added with 50 pmol/L miR-664-3p mimics and 10 μL Lipofectamine 2000), the inhibitor group (added with 50 pmol/L miR-664-3p inhibitors and 10 μL Lipofectamine 2000) and the negative control (NC) group (added with 50 pmol/L miR-664 NC and 10 μL Lipofectamine 2000). The cells were transfected in accordance to the instructions of Lipofectamine 2000 liposome transfection kit. Then they were placed in a thermostatic incubator with 5% CO_2_ at 37°C for incubation, and those in the logarithmic growth phase were applied for follow-up experiments. Primer sequences: miR-664-3p mimics: 5'-ACUGGCUAGGGA-AAAUGAUUGGAU-3'; miR-664-3p inhibitor: 5'-UGACCGAUCCCUUUUACUAACCUA-3'; miR-664-3p NC: 5'-CCUCCCUAGAACCCUGAAAGGUU-3'. Primers were designed and synthesized by Annoron (Beijing) Biotechnology Co., Ltd. RT-qPCR was used to assess transfection efficiency.

### Detection via qRT-PCR

The total RNA in tissues and cells were extracted using the TRIzol extraction kit. The optical density (OD) of RNA extracted was measured via an ultraviolet-visible spectrophotometer, and the integrity of the total RNA was determined by 1% agarose gel electrophoresis. Reverse transcription was conducted for complementary deoxyribonucleic acids (cDNAs) in a 20 μL reaction system based on M-MLV reverse transcriptase with the total RNA as a template. miR-664-3p fluorescence qPCR kits were adopted for detection on a fluorescence qPCR machine. The primer sequences are shown in Table 1. U6 was used as an internal reference. PCR conditions: predenaturation at 94°C for 1 min, denaturation at 95 °C for 15 s, annealing at 60°C for 20 s, extension at 72 °C for 1 min and re-extension at 72 °C for 10 min for a total of 35 cycles. At the end of these reactions, the cycle Ct value of each reaction tube was obtained. The relative expression level of miR-664-3p was relatively quantified using 2^−Δ^Ct^^ (13).

### Detection via Western Blot

Total protein was extracted from tissues and cells using the RIPA lysis method, and its concentration was detected with the bicinchoninic acid (BCA) method and adjusted to 4 μg/μL. The proteins were separated by 12% SDS-PAGE and then transferred to a polyvinylidene difluoride (PVDA) membrane. The membrane was stained with Ponceau S solution, immersed in PBST for 5 min and then washed, blocked with 5% skimmed milk powder for 2 h, and finally incubated overnight at 4°C with the primary antibodies (1:1000). Following washing to remove primary antibodies, the horse radish peroxidase-labeled goat anti-mouse secondary antibody (1:5000) was added to the membrane for a 1 h incubation at 37 °C. After that, the membrane was rinsed 3 times with TBST, for 5 min each time. The protein bands on the membrane were developed in a dark room using the enhanced chemiluminescence (ECL) reagent, and the excess liquid on the membrane was absorbed with a filter paper. The luminescent protein bands were scanned and the gray value was analyzed using Gel-Pro_analyzer 4.0. The relative expression level of each protein = the gray value of the target protein band / the gray value of the β-actin protein band.

### Detection of cell proliferation via MTT assay

The cells in logarithmic growth phase were prepared into 1×10^5^ cell suspension, and the cell density was adjusted to 2×10^4^ mL. The cells were inoculated in a 96-well plate at 2×10^3^/well. The cell viability was measured via MTT at the time of transfection for 24 h, 48 h, 72 h, 96 h and 120 h, respectively. During the measurement, 10 μL MTT solution (5 mg/mL) was added to each well, and the cells were further incubated in an incubator for 4 h. Each well was added with 150 μL formazan solving liquid and vibrated at room temperature for 10 min. The optical microscope revealed the complete dissolution of crystals, and the OD value at the wavelength of 570 nm was measured using a microplate reader. The detection for each well was repeated for 3 times.

### Detection of cell apoptosis via flow cytometry

After 48 h of transfection, cells were taken out and digested with trypsin. The collected cells were resuspended in cold 0.01 mol/L phosphate-buffered saline (PBS), and the cell count was 1×10^5^. After that, the cells were centrifuged at 1000 r/min for 5 min, after which the supernatant was discarded. Then the cells were resuspended in 150 μL Annexin V-FITC added and transferred to a flow cytometry tube. Each tube was added with 5 μL Annexin V-FITC and well mixed with 15 μL propidium iodide to each tube. The reaction endured 15 min at room temperature away from light, and the detection via a flow cytometer was completed within 1 h. Each specimen was repeatedly detected for 3 times.

### Statistical methods

Statistical Product and Service Solutions (SPSS) 20.0 (Beijing NDTimes Technology Co., Ltd.) was adopted for statistical analysis. Measurement data were expressed as mean ± standard deviation (x±sd). *t*-test was applied for comparisons of measurement data between groups, and the chi-square test was used for comparisons of count data between groups. Comparisons of the mean value among multiple groups were conducted using the one-way analysis of variance, after which the least significant difference (LSD)-*t* test was applied for pairwise comparisons. *P*<0.05 represented that the difference was statistically significant.

## Results

### Expressions of miR-664-3p in osteosarcoma tissues and normal bone tissues

**Fig. 2:**
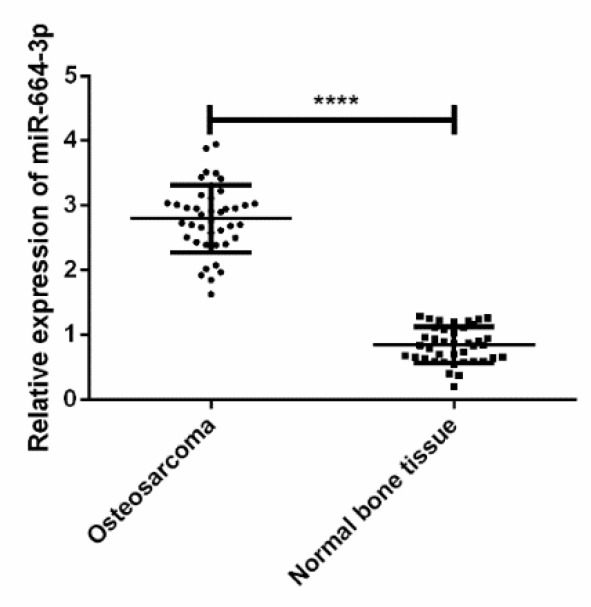
Expressions of miR-664-3p in osteosarcoma tissues and normal bone tissues. The expression level of miR-664-3p in osteosarcoma tissues was significantly higher than that in normal bone tissues (*t*=19.680, *P*<0.001). Note: ^****^P<0.001

The expression level of miR-664-3p in osteosarcoma tissues was significantly higher than that in normal bone tissues (*t*=19.680, *P*<0.001) (Fig. 2).

### Expression of microRNA-664-3p in osteosarcoma tissue in patients with clinicopathological parameters

There was no significant difference in the expression of miR-664-3p in osteosarcoma tissues in clinical pathology, such as gender, age, grade of malignancy, pathological type, differentiation degree and so on (Table 2).

**Table 2:** Expression of microRNA-664-3p in osteosarcoma tissue in patients with clinicopathological parameters (x±sd)

***Item***	***n***	***miR-664-3p***	**P *value***
Gender			0.281
Male	22	2.613±0.496	
Female	19	2.797±0.584	
Age (yr)			0.542
≤15	21	2.816±0.634	
>15	20	2.709±0.464	
Malignancy grade			0.473
Grade 1	10	2.547±0.347	
Grade 2	16	2.704±0.573	
Grade 3	15	2.834±0.674	
Pathological type			0.630
Osteoblast type	9	2.634±0.449	
Chondroblast type	10	2.549±0.613	
Fibroblast type	14	2.749±0.634	
Mixed type	8	2.894±0.602	
Degree of pathological differentiation			0.897
High differentiation	8	2.768±0.550	
Moderate differentiation	22	2.742±0.567	
Low differentiation	11	2.836±0.497	

### Expression of miR-664-3p in osteosarcoma cells

**Fig. 3:**
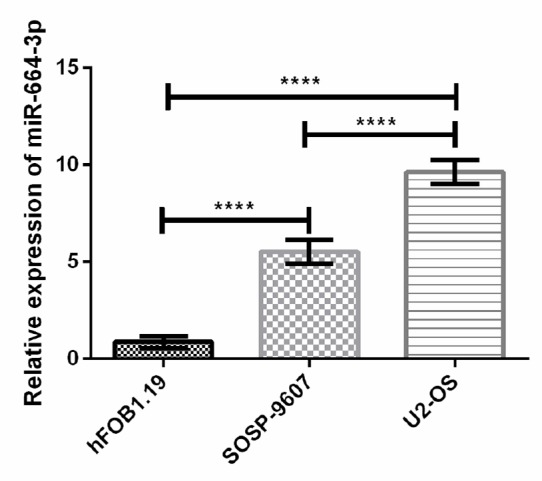
Expression of miR-664-3p in osteosarcoma cells. the relative expression levels of miR-664-3p in SOSP-9607 and U2-OS were significantly higher than that in hFOB1.19. The relative expression level of miR-664-3p in U2-OS was significantly higher than that in SOSP-9607 (*P*<0.001) Note: ^****^P<0.001

According to qRT-PCR results, the relative expression levels of miR-664-3p in SOSP-9607 and U2-OS were significantly higher than that in hFOB1.19 (*P*<0.001). The relative expression level of miR-664-3p in U2-OS was significantly higher than that in SOSP-9607 (*P*<0.001) (Fig. 3).

### Expression of miR-664-3p in U2-OS cells after transfection

**Fig. 4:**
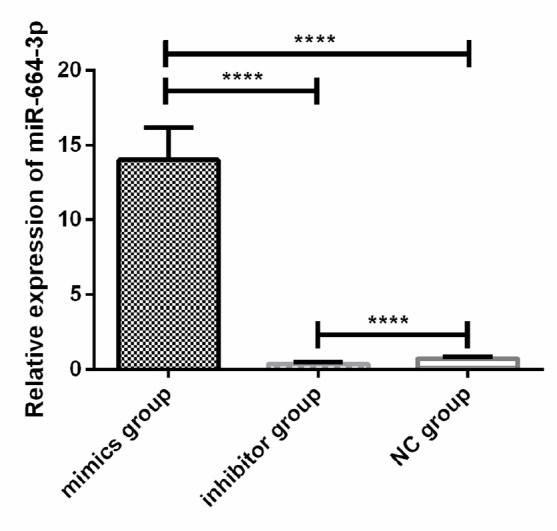
Expression of miR-664-3p in U2-OS cells after transfection The results of qRT-PCR showed that the relative expression of miR-664-3p in U2-OS cells in the mimics group was significantly higher than that in the inhibitor group and the NG group (*p*<0.001), and the relative expression of miR-664-3p in U2-OS cells in the NG group was significantly higher than that in the inhibitor group (*P*<0.001). Note: ^****^P<0.001

The results of qRT-PCR showed that the relative expression of miR-664-3p in U2-OS cells in the mimics group was significantly higher than that in the inhibitor group and the NG group (*P*<0.001), and the relative expression of miR-664-3p in U2-OS cells in the NG group was significantly higher than that in the inhibitor group (*P*<0.001)(Fig. 4).

### Effects of the expression of miR-664-3p on the proliferation of osteosarcoma cell line U2-OS

**Fig. 5:**
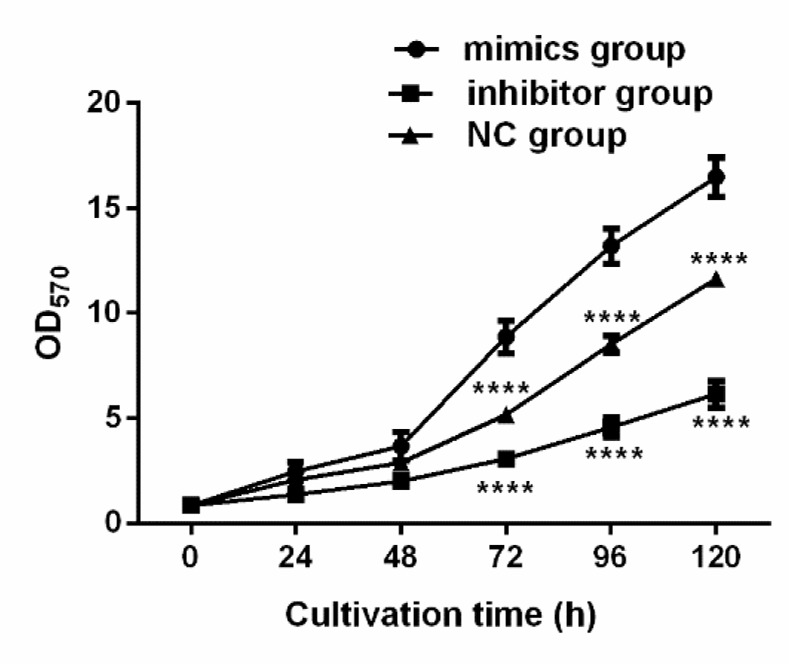
Effects of miR-664-3p expression on the proliferation of osteosarcoma cell line U2-OS. The results of MTT assay manifest that the OD value detected in the mimics group is obviously higher than that in NC group from 48 h (*P*<0.001). The OD value detected in the inhibitor group is significantly lower than those in the mimics group and the NC group from 48 h (*P*<0.001). Note: ^****^P<0.001 vs. mimics group

MTT assay was adopted to detect the effects of miR-664-3p at different time of transfection on the proliferation of osteosarcoma cell line U2-OS. The results revealed that the OD value detected in the mimics group was significantly higher than that in the NC group from 48 h (*P*<0.001), and the value in the inhibitor group was evidently lower than those in the mimics group and NC group from 48 h (*P*<0.001) (Fig. 5).

### Effects of the expression of miR-664-3p on the apoptosis of osteosarcoma cell line U2-OS

**Fig. 6:**
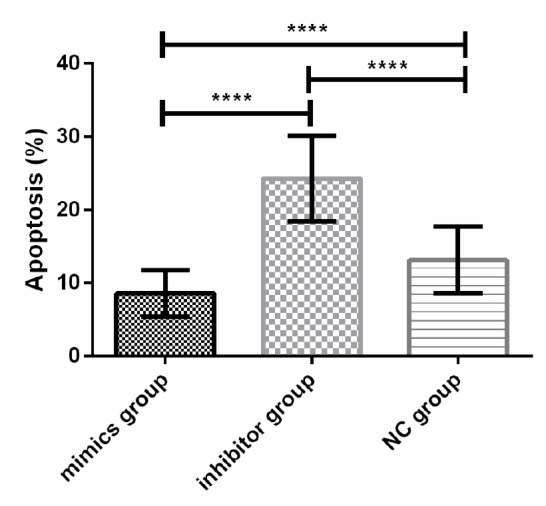
Effects of the expression of miR-664-3p on the apoptosis of osteosarcoma cell line U2-OS. The results of flow cytometry indicate that the total apoptosis rate in the inhibitor group is significantly higher than those in the mimics group and the NC group The total apoptosis rate in the mimics group is significantly lower than that in the NC group (*P*<0.001). Note: ^****^P<0.001

The results of flow cytometry manifested that the total apoptosis rate in the inhibitor group was significantly higher than that in the mimics group and the NC group (*P*<0.001), and the total apoptosis rate in the mimics group was significantly lower than that in the NC group (*P*<0.001) (Fig. 6).

### Effects of the expression of miR-664-3p on the expressions of PLP2 in osteosarcoma cell line U2-OS

**Fig. 7:**
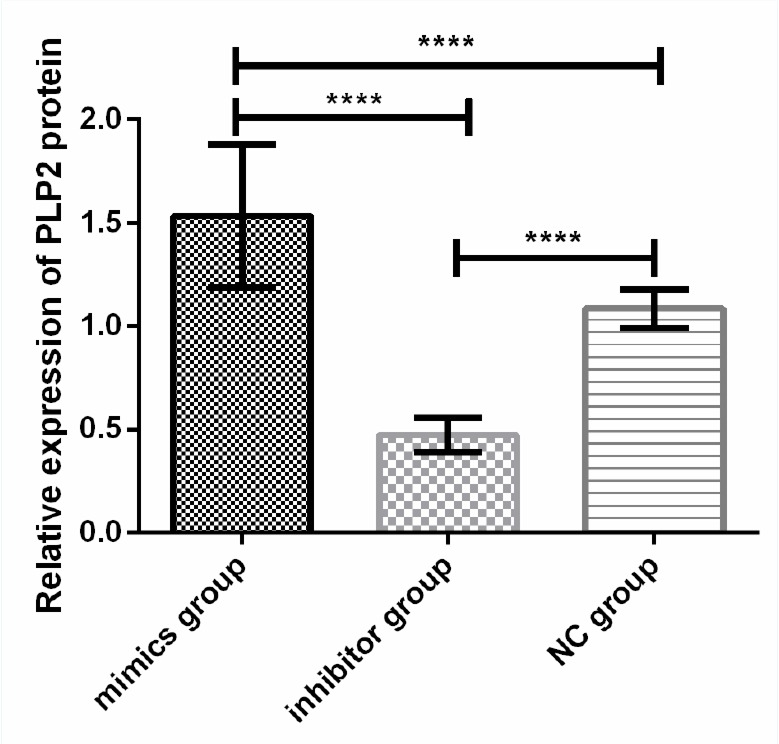
Effects of the expression of miR-664-3p on the expression of PLP2 in osteosarcoma cell line U2-OS. Western Blot results reveal that the relative expression level of PLP2 in the inhibitor group is obviously lower than those in the mimics group and the NC group. The relative expression level of PLP2 in the mimics group is remarkably higher than those in the NC group (*P*<0.001). Note: ^****^P<0.001

qRT-PCR was adopted to detect the effects of the expression of miR-664-3p after transfection on PLP2 in U2-OS. The results showed that the relative expression level of PLP2 in the inhibitor group was significantly lower than that in the mimics group and the NC group (*P*<0.001), and the level in the mimics group was significantly higher than that in the NC group (*P*<0.001) (Fig. 7).

## Discussion

Osteosarcoma is a type of malignant tumor characterized by strong local invasiveness (14, 15). According to a study, osteosarcoma patients with metastasis have strong resistance to chemotherapeutics, and approximately 30% of patients have no sensitivity to chemotherapeutics, which leads to poor clinical prognosis (16). The mechanisms of the occurrence and development of osteosarcoma have not been clarified, but the proliferation and apoptosis of tumor cells are one of the mechanisms of osteosarcoma (17).

Hence, it is of great significance to investigate the pathogenic mechanism of osteosarcoma and explore molecular biologic markers and therapeutic targets closely associated with the occurrence and development of osteosarcoma for the diagnosis of patients with osteosarcoma and the treatment of cancer molecules.

Regulating the vital process of organisms, miRNAs regulate gene expression during or after transcription, so as to be involved in multiple physiological and pathological processes, such as organ development, cell differentiation, proliferation, apoptosis, cancerization and metabolism (18–20). miR-664-3p plays an important role in many diseases. For example, acarbose could improve blood glucose in diabetic rats through MAPK pathway and down-regulate pro-inflammatory factors by activating miR-10a-5p and miR-664 in ileum (21). miR-664 promoted cell proliferation in T cell acute lymphoblastic leukemia (T-ALL) cell lines (22). It was found in this study that the relative expression level of miR-664-3p in osteosarcoma tissues was significantly higher than that in normal bone tissues, indicating that miR-664-3p may exert a crucial effect in the occurrence and development of osteosarcoma. miR-664-3p was further transiently transfected in vitro, which manifested that the OD value in the mimics group was significantly higher than that in the NC group from 48 h, while the value in the inhibitor group was obviously lower than that in the NC group from 48 h. In addition, the total apoptosis rate in the inhibitor group was significantly higher than those in the mimics group and the NC group, and the total apoptosis rate in the mimics group was significantly lower than that in the NC group, suggesting that miR-664-3p over-expression can significantly suppress the proliferation of osteosarcoma cell line U2-OS and promote its apoptosis, while the inhibition of miR-664-3p expression plays an opposite role. miR-664-3p, therefore, may be regarded as a biological indicator for novel targeted therapy of osteosarcoma.

In order to further analyze the regulatory mechanism of miR-664-3p on proliferation and apoptosis in U2-OS cells, a target gene prediction analysis of miR-664-3p was carried out, and it was found that PLP2 may be a potential target gene of miR-664-3p. PLP2 is significantly up-regulated in a variety of tumors, including breast cancer, hepatocellular carcinoma, osteosarcoma, and melanoma (23, 24). In this study, the relative expression level of PLP2 in the inhibitor group was significantly lower than that in the mimics group and the NC group, and the level in the mimics group was significantly higher than that in the NC group, which indicates that miR-664-3p may play a role in the proliferation and apoptosis of osteosarcoma cells by regulating the expression of PLP2. miR-664 could promote the proliferation and invasion of T-ALL by negatively regulating PLP2 (25). PLP2 was a direct target for miR-664 in cutaneous malignant melanoma (CMM) cells (26). Therefore, miR-664-3p is of great biological significance by directly targeting PLP2 in tumors. However, the different regulatory mechanisms caused by the different growth and invasion patterns of various types of tumors need to be further studied and analyzed.

The effects of miR-664-3p after transfection on the proliferation and apoptosis of osteosarcoma cells were investigated in this study, so as to observe its regulatory effect in the process, thus providing a theoretical basis for the clinical treatment of osteosarcoma. However, there are still limitations in the experimental design. Firstly, the relationship between miR-664-3p and PLP2 has not been confirmed through the dual luciferase reporter assay. Secondly, the effects of miR-664-3p on the migration and invasion of osteosarcoma cell line U2-OS were not studied, and the regulatory mechanism remains unclear. Therefore, more studies are needed in the future to support the results of this study.

## Conclusion

miR-664-3p may be involved in the occurrence and development of osteosarcoma, and can regulate the proliferation and apoptosis of U2-OS cells, and the expression of PLP2. Besides, miR-664-3p may become a novel molecular biological indicator for the diagnosis, targeted treatment and prognosis assessment of osteosarcoma.

## Ethical considerations

Ethical issues (Including plagiarism, informed consent, misconduct, data fabrication and/or falsification, double publication and/or submission, redundancy, etc.) have been completely observed by the authors.
